# Supplementary material for: Geographic Provenances Outweigh Tissue Compartments in Bacteriome Assembly of the Ectomycorrhizal, Edible, and Hallucinogenic if Undercooked, Lanmoa asiatica (Boletaceae, Boletales) Mushroom from Yunnan China
Source: Microorganisms. 2025 Oct 23;13(11):2431. doi: 10.3390/microorganisms13112431 (PMC12654045; doi:10.3390/microorganisms13112431)
Supplement: Supplementary file 1 [file microorganisms-13-02431-s001.zip › microorganisms-3907434-supplementary.pdf]

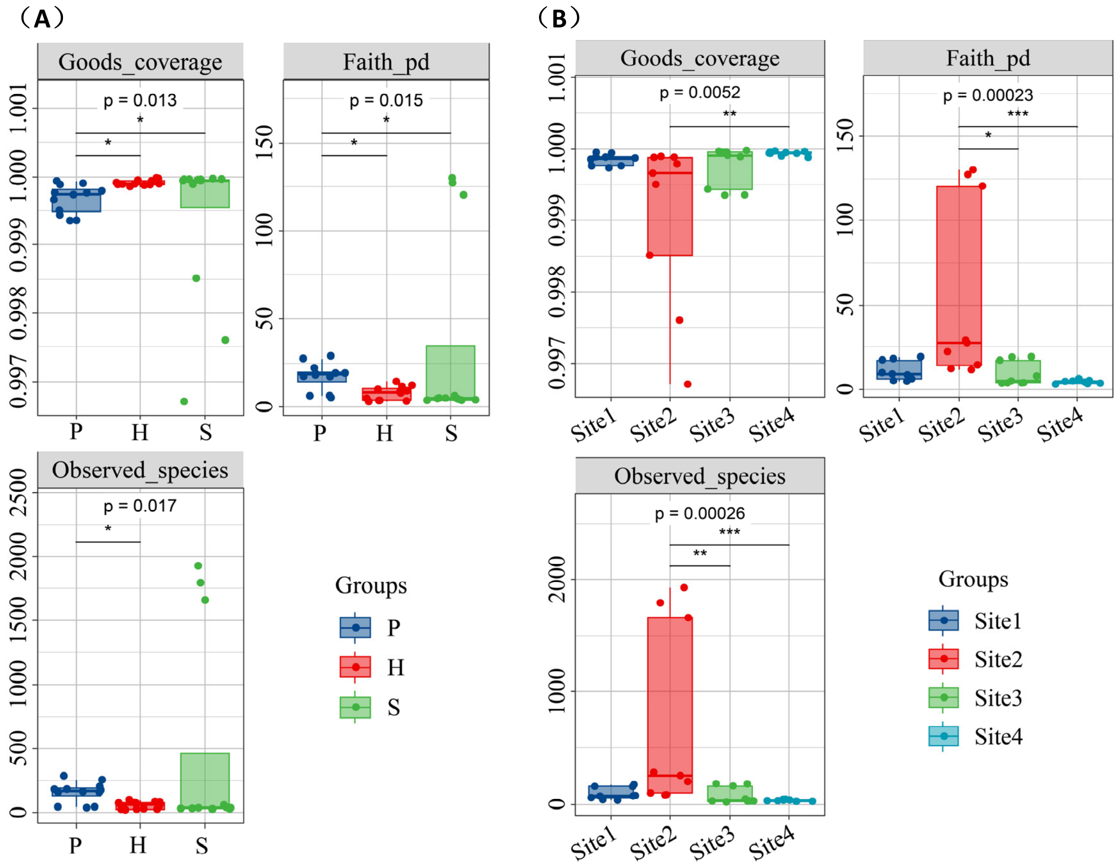


**Supplementary Figure S1.** Alpha diversity of endophytic bacteria within *Lanmoa asiatica* basidiomata across anatomical sections (A) and geographical locations (B). Boxplots depict variations in diversity indices. Horizontal lines above the boxplots denotate statistically significant pairwise comparisons derived from t-tests; asterisks indicate significance levels (*p < 0.05, **p < 0.01). Dark horizontal lines connect groups exhibiting significant differences in mean values. Abbreviations for basidiomata tissues (left) and geographic sampling locations (right) correspond to those specified in Figure 3 in the manuscript text.

**Supplementary Table S1.** Taxonomic classification of the core endophytic bacterial taxa, defined as those operational taxonomic units (OTUs) present in all three compartment tissues (hymenophore, pileus context, stipe context) of *Lanmoa asiatica* basidiomata.

| #ID | Taxonomy of the shared bacteria |
|---|---|
| ASV_66 | o__Sphingomonadales; f__Sphingomonadaceae; *g__Novosphingobium; s__unclassified_Novosphingobium* |
| ASV_77 | o__Enterobacterales; f__Erwiniaceae; *g__Pantoea; s__unclassified_Pantoea* |
| ASV_253 | o__Staphylococcales; f__Staphylococcaceae; *g__Staphylococcus; s__unclassified_Staphylococcus* |
| ASV_474 | o__Lactobacillales; f__unclassified_Lactobacillales; *g__unclassified_Lactobacillales; s__unclassified_Lactobacillales* |
| ASV_476 | o__Rhizobiales; f__Beijerinckiaceae; *g__uncultured; s__uncultured_bacterium* |
| ASV_530 | o__Bacillales; f__Bacillaceae; *g__Bacillus; s__unclassified_Bacillus* |
| ASV_550 | o__Enterobacterales; f__Erwiniaceae; *g__Pantoea; s__unclassified_Pantoea* |
| ASV_555 | o__Sphingomonadales; f__Sphingomonadaceae; *g__Sphingomonas; s__unclassified_Sphingomonas* |
| ASV_655 | o__Elsterales; f__Elsteraceae; *g__uncultured; s__uncultured_bacterium* |
| ASV_1152 | o__Rhizobiales; f__Xanthobacteraceae; *g__Bradyrhizobium; s__unclassified_Bradyrhizobium* |

| | |
|---|---|
| ASV_1193 | o__Enterobacterales; f__Yersiniaceae; *g__Serratia; s__unclassified_Serratia* |
| ASV_1493 | o__Lactobacillales; f__Lactobacillaceae; *g__Leuconostoc; s__unclassified_Leuconostoc* |
| ASV_1537 | o__Lactobacillales; f__Aerococcaceae; *g__Facklamia; s__uncultured_bacterium* |
| ASV_1714 | o__Enterobacterales; f__Erwiniaceae; *g__Pantoea; s__unclassified_Pantoea* |
| ASV_1897 | o__Staphylococcales; f__Staphylococcaceae; *g__Staphylococcus; s__unclassified_Staphylococcus* |
| ASV_1928 | o__Enterobacterales; f__Yersiniaceae; *g__Serratia; s__Ewingella_americana* |
| ASV_2284 | o__Burkholderiales; f__Burkholderiaceae; *g__Burkholderia-Caballeronia-Paraburkholderia; s__Caballeronia_sordidicola* |
| ASV_2470 | o__Myxococcales; f__Anaeromyxobacteraceae; *g__Anaeromyxobacter; s__uncultured_bacterium* |
| ASV_2662 | o__Elsterales; f__uncultured; *g__uncultured; s__unclassified_uncultured* |
| ASV_2926 | o__Rhodobacterales; f__Rhodobacteraceae; *g__Paracoccus; s__Paracoccus_marcusii* |
| ASV_2930 | o__Rhizobiales; f__Beijerinckiaceae; *g__Roseiarcus; s__uncultured_bacterium* |
| ASV_3107 | o__Sphingomonadales; f__Sphingomonadaceae; *g__Sphingomonas; s__unclassified_Sphingomonas* |
| ASV_3180 | o__Frankiales; f__Acidothermaceae; *g__Acidothermus; s__unclassified_Acidothermus* |
| ASV_3804 | o__Caulobacterales; f__Caulobacteraceae; *g__Brevundimonas; s__unclassified_Brevundimonas* |
| ASV_3864 | o__Sphingomonadales; f__Sphingomonadaceae; *g__Novosphingobium; s__unclassified_Novosphingobium* |
| ASV_4109 | o__Pseudomonadales; f__Pseudomonadaceae; *g__Pseudomonas; s__unclassified_Pseudomonas* |
| ASV_4747 | o__Solirubrobacterales; f__Solirubrobacteraceae; *g__uncultured; s__uncultured_Conexibacteraceae* |
| ASV_4850 | o__Acetobacterales; f__Acetobacteraceae; *g__unclassified_Acetobacteraceae; s__unclassified_Acetobacteraceae* |
| ASV_4895 | o__Pseudomonadales; f__Nitrincolaceae; *g__Marinobacterium; s__Marinobacterium_sp.* |
| ASV_5341 | o__Acetobacterales; f__Acetobacteraceae; *g__uncultured; s__metagenome* |
| ASV_5386 | o__Pseudomonadales; f__Pseudomonadaceae; *g__Pseudomonas; s__unclassified_Pseudomonas* |
| ASV_6039 | o__Rhizobiales; f__Beijerinckiaceae; *g__Roseiarcus; s__uncultured_bacterium* |
| ASV_6411 | o__Acetobacterales; f__Acetobacteraceae; *g__Acidiphilium; s__unclassified_Acidiphilium* |
| ASV_6522 | o__Burkholderiales; f__Burkholderiaceae; *g__Cupriavidus; s__unclassified_Cupriavidus* |
| ASV_6923 | o__Acetobacterales; f__Acetobacteraceae; *g__uncultured; s__unclassified_uncultured* |
| ASV_6946 | o__Caulobacterales; f__Caulobacteraceae; *g__uncultured; s__unclassified_uncultured* |
| ASV_7006 | o__Acetobacterales; f__Acetobacteraceae; *g__uncultured; s__unclassified_uncultured* |

**Supplementary Table S2.** Taxonomic classification of the core endophytic bacterial taxa, defined as those operational taxonomic units (OTUs) present in four geographical locations of *Lanmoa asiatica* basidiomata. Detailed geographic information of the provenances correspond to those specified in Figure 3 in the manuscript text.

| #ID | Taxonomy of the shared bacteria |
|---|---|
| ASV_1493 | o__Lactobacillales; f__Lactobacillaceae; *g__Leuconostoc; s__unclassified_Leuconostoc* |
| ASV_1560 | o__Staphylococcales; f__Staphylococcaceae; *g__Staphylococcus; s__unclassified_Staphylococcus* |
| ASV_1714 | o__Enterobacterales; f__Erwiniaceae; *g__Pantoea; s__unclassified_Pantoea* |
